# Beyond the Pandemic: The Value of Antimicrobial Stewardship

**DOI:** 10.3389/fpubh.2022.902835

**Published:** 2022-06-27

**Authors:** Souha S. Kanj, Paula Ramirez, Camilla Rodrigues

**Affiliations:** ^1^Division of Infectious Diseases, American University of Beirut Medical Center, Beirut, Lebanon; ^2^Critical Care Department, Hospital Universitari I Politècnic La Fe, Valencia, Spain; ^3^Department of Microbiology, P. D. Hinduja Hospital and Medical Research Centre, Mumbai, India

**Keywords:** COVID-19, stewardship, antimicrobial resistance, infection prevention and control, One Health, lessons learned

## Introduction

The COVID-19 pandemic has highlighted critical weaknesses in health, surveillance, and reporting systems around the world, and has diverted antimicrobial stewardship (AMS) efforts (1, 2). However, it has offered vital lessons that can be applied to counteract the ongoing threat of antimicrobial resistance (AMR). The importance and role of AMS and global coordination of efforts against AMR cannot be overemphasized with respect to implementation of new stewardship programs and strengthening of existing ones; improving access to diagnostics and laboratory equipment; the need for development and dissemination of evidence-based guidelines for appropriate antimicrobial use; continued education of all stakeholders and strengthening of the One Health Approach. Coordinated strategies as part of AMS go hand-in-hand with infection prevention and control (IPC) and microbial surveillance (3).

### Multidrug-Resistant Infections in COVID-19 Patients and Inappropriate Prescribing of Antimicrobial Therapy

The decision to initiate empirical antibiotic therapy in COVID-19-infected patients is complicated by the fact that the clinical, radiological, and laboratory signs of severe COVID-19 infection can mimic those of bacterial pneumonia (4). This means many patients with COVID-19 receive empirical antibiotic treatment inappropriately (4, 5). In patients with suspected bacterial pneumonia and/or septic shock, international guidelines recommend the prompt use of empiric, broad-spectrum antibiotics prior to microbiological test results based on clinical judgment (6, 7). Conversely, culture-based approaches are recommended to limit overdiagnosis and facilitate appropriate antimicrobial therapy in ventilator-associated pneumonia (VAP) (7–9). A study of hospitalized COVID-19 patients in New York found that 60% of patients had positive respiratory cultures and 54% had positive blood cultures, but multidrug-resistant (MDR) Gram-negative isolates were present in just 15 and 8.5% of cultures, respectively. As many as 79% of patients included in the study had antibiotic exposure prior to testing, increasing to 98% at some time point during their COVID-19 hospitalization. All patients with identified MDR organisms had previous antibiotic exposure, compared to 65% of patients without MDR organisms (10).

Since the start of the pandemic, an unprecedented number of patients have been admitted to intensive care units (ICU) with COVID-19-related severe respiratory failure, requiring invasive mechanical ventilation (IMV) (11). Extended hospitalization and IMV are both known risk factors for acquiring infections with MDR pathogens. A systematic review and meta-analysis of studies from the United Kingdom, Europe, Russia, and China showed that patients with COVID-19 are at significantly greater risk of VAP than those intubated but without COVID-19, with a pooled estimated mortality rate in co-infected patients of 42.7% (12). The use of extracorporeal membrane oxygenation in COVID-19 patients with reversible respiratory failure also poses a significant risk for secondary infection (13). *Enterobacterales* spp., *Pseudomonas aeruginosa*, and *Acinetobacter baumannii* have been among the most reported MDR bacteria isolated from superinfected patients, limiting the available choices of therapy (12–17).

Spikes in secondary fungal infections have also been noted worldwide. Mucormycosis, known to affect immunocompromised patients, in particular those with uncontrolled diabetes, was responsible for a significant increase in cases of co-infection in India (18). COVID-19-associated pulmonary aspergillosis has been reported in many case series, including in previously healthy COVID-19-infected patients. In addition, *Candida auris* is emerging as a global threat, with reports from countries which had never isolated *C. auris* prior to the pandemic (4, 19, 20). Whether inappropriate antibiotic use contributed to the rise of these fungal infections remains to be determined.

### Compromised Healthcare Services

Disruptions in screening and surveillance (2, 21, 22) of MDR pathogens, reduced AMS activities (2, 22), lack of proper isolation of patients infected with MDR pathogens, and increased use of broad-spectrum antimicrobial agents have all contributed to increases in AMR (23). Increased pressure on healthcare services during the pandemic has lowered the priority and accessibility of laboratory diagnostics in some hospitals, or exacerbated pre-existing lack of diagnostic capability, as well as diverting the efforts of infection control practitioners in most settings to COVID-19 prevention and management. An informal 2020 Twitter poll showed that 79.1% of hospital IPC personnel spent more than 75% of their time on COVID-19 response efforts–a diversion from their traditional infection prevention tasks. A survey of 73 countries across the World Health Organization (WHO) Global Antimicrobial Resistance and Use Surveillance System (GLASS) network found that between October and December 2020, 67% reported limited ability to work with AMR partnerships; reduced funding was frequently reported by low- and middle-income countries (LMIC); reduced availability of nursing, medical, and public health staff for AMR was reported by 71, 69, and 64%, respectively, whereas 67% reported stable housekeeping staff availability. The majority (58%) reported reduced reagents/consumables, particularly those from LMICs (24).

### Poor Regulation of Antimicrobial Distribution and Use in LMICs

Clinicians in LMICs may find it difficult to access evidence-based antimicrobial prescribing guidelines, which may drive further AMR. Poor regulation of the distribution and use of antimicrobials has resulted in antibiotics being easily purchased over the counter in many LMICs. COVID-19 has impacted the availability of antimicrobials due to the disruption of supply chains and global manufacturing, which, in turn, has led to changes in antimicrobial usage patterns (1, 25).

Poor healthcare system structure and limited antimicrobial stewardship programs (ASP), which are often linked to lack of resources, such as trained personnel and under-equipped diagnostic laboratories, are also important drivers of AMR in these regions (1, 23, 26).

## Discussion

### AMS Is Essential to Curtail Inappropriate Use of Antimicrobial Agents

In the context of inevitable future threats to our healthcare systems, communication on inappropriate/appropriate treatment regimens must be improved and made timelier (24). Use of collected data on patients and AMR surveillance to inform correct management of secondary bacterial infections and sepsis should be viewed as vital. To this end, a true multidisciplinary effort is warranted. The literature has shown us time and time again that when hospital-based multidisciplinary teams which involve all levels of staff in the treatment pyramid work effectively together, patient outcomes are improved across numerous parameters (27). Moreover, when multidisciplinary teams operate within a stewardship program, a positive bearing on the perception, attitudes, and practices of hospital doctors toward the management of patients with multidrug-resistant infections is demonstrated (28).

### AMR Surveillance and Diagnostics Must Be Maintained During the Ongoing COVID-19 Pandemic

Accurate diagnostic and surveillance systems are essential to tackling the threat of AMR because they ensure appropriate selection of antimicrobial agents and allow the monitoring of the impact of ASPs on AMR. Moreover, access to diagnostics is key to the achievement of the United Nations Sustainable Development Goal of universal health coverage (23, 29).

Lower rates of COVID-19 testing, lack of trained laboratory staff to carry out COVID-19 analysis in LMICs vs. higher-income countries, and the limited participation of LMICs in the WHO's GLASS surveillance program for AMR monitoring have highlighted the urgent need for access to diagnostics and training for laboratory personnel in these regions (23, 30–32). In the context of COVID-19, the WHO rapidly called for research into point-of-care (PoC) diagnostics for use at community level (33). These efforts must also extend to the development, funding, and implementation of rapid bacterial infectious disease and PoC testing (PoCT) to fight AMR (23). This would also facilitate a “pick-and-mix” implementation strategy for PoCT that can be tailored to meet the needs of individual stakeholders across countries (including resource-limited regions) who are involved in the development and use of rapid diagnostics (23, 34). Improved availability of surveillance and diagnostic systems (including PoCT) in LMICs would then translate to more appropriate antimicrobial prescribing in these regions (35, 36).

### IPC Cannot Be Compromised in the Fight Against AMR

The COVID-19 pandemic reinforced the importance and raised the status of IPC (24). Indeed, many respondent countries across the GLASS network reported improvements in IPC activities because of COVID-19 (24).

However, there were also reports of inappropriate IPC practices. Low-, middle-, and high-income countries have all highlighted the need to strengthen IPC efforts through awareness campaigns and training, and those at higher income levels also noted the need for better integration of IPC across entire healthcare systems. The current pandemic offers an opportunity to implement more effective IPC programs that can withstand and combat future emerging threats by (24):

- Promoting evidence-based IPC guidance on appropriate standard and transmission-based precautions, with local adaptation.
- Educating to prevent inappropriate IPC practices (e.g., incorrect use of personal protective equipment).
- Taking the opportunity to advocate for more sustainable IPC programs beyond the duration of outbreaks, so that we can combat future emerging threats more effectively (37).

### Education and Awareness Are Needed to Drive Positive Behavioral Change Toward the Use of Antimicrobials

There should be continuous educational campaigns to educate healthcare workers and patients on the need for appropriate prescribing and use of antimicrobial medications, including antivirals and antifungal medications (1, 23, 38). Such campaigns targeting the public and healthcare workers have been organized and implemented using different approaches and methodologies, although mainly by high-income countries (39). Clinician-directed interventions include educational materials, audit and feedback on antibiotic prescribing practices, electronic or paper reminders, and computer-aided clinical decision support systems (40). Social media and gamification have the capacity to deliver health education and convey useful information to the public and have been successfully used in developed countries. Increase in the use of mobile phones and social media in LMICs presents an opportunity to increase awareness about disease prevention in a rapid, engaging, and cost-effective way (41).

### AMS Programs and Healthcare Systems Need to Be Stronger and More Prepared

Reductions in resourcing and the routine ability to work with partnerships could lead to gaps in communication, AMS implementation, and AMR data exchange. Although programmatic and structural factors that could reduce AMR have been reported by some countries, others were able to identify areas where the COVID-19 and AMR responses overlapped, leading to the development of integrated stewardship guidance, new partnership platforms, leverage of funding for healthcare-system strengthening, and improvements in laboratory networks (24).

In addition to the identification of overlaps between the COVID-19 and AMR response, the following are critical to maintaining AMR control during the COVID-19 era and in the event of future threats (24):

- Re-establishment of partnerships (and the development of new ones) and training programs, once the local situation allows.
- Recognition of where individual COVID-19 and AMR efforts are needed (e.g., investment in workforce development).
- Rapid communication and data sharing within AMR surveillance networks and to other relevant stakeholders to mitigate future threats.
- Update of national AMR action plans with best practice approaches to prepare for emerging threats.
- Broader system strengthening using multidisciplinary teams (i.e., clinicians, epidemiologists, lab scientists, data managers) to develop communities of practice.
- Continued international engagement to advocate AMR as an ongoing global health priority.

### AMS Needs a Global, Integrated One Health Approach to Move Beyond Pandemic Preparedness

The COVID-19 pandemic has emphasized the need for significant global collaboration between governments and healthcare teams to tackle the greatest threat to public health to date (42). However, not all governments have responded optimally (1, 43–45). Attention is now being focused on preparedness vs. response, *via* improved IPC strategies and the integration of ASPs into “disaster preparedness plans” (46). In the wake of an increased understanding of the reciprocity of human and animal health following the SARS-CoV-1 and H1N1 influenza pandemics, including the potential impact of zoonoses on AMR, the One Health concept has gained interest (47).

The WHO Global Action Plan to address the AMR crisis embraces a One Health approach and calls on member states to consider the following when developing their own action plans (48, 49):

1. Improve awareness and understanding of AMR through effective communication, education, and training.
2. Strengthen the knowledge and evidence base through surveillance and research.
3. Reduce the incidence of infection through effective sanitation, hygiene, and IPC measures.
4. Optimize the use of antimicrobial medicines in human and animal health.
5. Develop the economic case for sustainable investment that considers the needs of all countries, and increase investment in new medicines, diagnostic tools, vaccines, and other interventions.

Coordination and solidarity will be critical in tackling AMR on a regional and international scale (47, 50). A collaborative, cross-sector, multidisciplinary approach to AMS that engages healthcare leaders, microbiologists, infectious disease specialists, physicians, nurses, farmers, veterinarians, IT experts, and clinical pharmacists will improve patient treatment outcomes and safety by reducing AMR development (51–53). However, there are barriers to overcome, including competing interests among multiple sectors and organizations, lack of consensus on priorities for action, and gaps in AMR surveillance, antimicrobial use policy, and IPC in many global regions (48). Ongoing and future efforts must build on successful international initiatives (e.g., tripartite meeting scheme between the EU, the US, and Japan, Transatlantic Task Force on Antimicrobial Resistance, and the Codex Alimentarius Task Force on Antimicrobial Resistance) (50).

The COVID-19 pandemic has exacerbated the threat of AMR, but it is not too late to mitigate this pandemic within a pandemic. Lessons learned from the past 2 years have given us the chance to strengthen global AMS efforts moving forwards. But we must act now.

## Author Contributions

SK, PR, and CR developed the concept, authored the paper, and made substantial contributions to the drafts including revising it critically for important intellectual content. All authors gave their final approval of the version to be published.

## Funding

Medical writing support was funded by Pfizer in accordance with Good Publication Practice (GPP3) guidelines.

## Conflict of Interest

SK has received honoraria from MSD, Pfizer, Gilead, and Novartis for participation in speaker activities and advisory boards, and for written contributions to UpToDate. CR has received honoraria from Pfizer, Sanofi, BioMérieux, Becton Dickinson, Cipla, Glenmark, Novartis, MSD, and Alkem for speaker activities and advisory boards. PR has received honoraria from MSD, Pfizer, Gilead, Shionogi, Menarini, and Novartis for participation in speaker activities and advisory boards.

## Publisher's Note

All claims expressed in this article are solely those of the authors and do not necessarily represent those of their affiliated organizations, or those of the publisher, the editors and the reviewers. Any product that may be evaluated in this article, or claim that may be made by its manufacturer, is not guaranteed or endorsed by the publisher.
